# Emergence of sporadic non-clustered cases of hospital-associated listeriosis among immunocompromised adults in southern Taiwan from 1992 to 2013: effect of precipitating immunosuppressive agents

**DOI:** 10.1186/1471-2334-14-145

**Published:** 2014-03-19

**Authors:** Chun-Yuan Lee, Hung-Chin Tsai, Calvin M Kunin, Susan Shin-Jung Lee, Kuan-Sheng Wu, Yao-Shen Chen

**Affiliations:** 1 Division of Infectious Diseases, Department of Medicine, Kaohsiung Veterans General Hospital, 386 Ta-Chung 1st Rd., Kaohsiung 813, Taiwan; 2 Faculty of Medicine, School of Medicine, National Yang-Ming University, Taipei, Taiwan; 3 Department of Internal Medicine (CMK), Ohio State University, Columbus, Ohio and the University of Arizona, Tucson, Arizona, USA; 4 Graduate Institute of Science Education and Environmental Education, National Kaohsiung Normal University, Kaohsiung, Taiwan

**Keywords:** Immunocompromised host, *Listeria monocytogenes*, Hospital-associated infection

## Abstract

**Background:**

Sporadic non-clustered hospital-associated listeriosis is an emerging infectious disease in immunocompromised hosts. The current study was designed to determine the impact of long-term and precipitating immunosuppressive agents and underlying diseases on triggering the expression of the disease, and to compare the clinical features and outcome of hospital-associated and community-associated listeriosis.

**Methods:**

We reviewed the medical records of all patients with *Listeria monocytogenes* isolated from sterile body sites at a large medical center in southern Taiwan during 1992–2013. Non-clustered cases were defined as those unrelated to any other in time or place. Multivariable regression analysis was used to determine factors associated with prognosis.

**Results:**

Thirty-five non-clustered cases of listeriosis were identified. Twelve (34.2%) were hospital-associated, and 23 (65.7%) were community-associated. The 60-day mortality was significantly greater in hospital-associated than in community-associated cases (66.7% vs. 17.4%, *p* = 0.007). Significantly more hospital-associated than community-associated cases were treated with a precipitating immunosuppressive agent within 4 weeks prior to onset of listeriosis (91.7% vs. 4.3%, respectively *p* < 0.001). The median period from the start of precipitating immunosuppressive treatment to the onset of listeriosis-related symptoms was 12 days (range, 4–27 days) in 11 of the 12 hospital-associated cases. In the multivariable analysis, APACHE II score >21 (*p* = 0.04) and receipt of precipitating immunosuppressive therapy (*p* = 0.02) were independent risk factors for 60-day mortality.

**Conclusions:**

Sporadic non-clustered hospital-associated listeriosis needs to be considered in the differential diagnosis of sepsis in immunocompromised patients, particularly in those treated with new or increased doses of immunosuppressive agents.

## Background

*Listeria monocytogenes* is an important cause of disease in humans, particularly in patients with impaired cellular immunity [1]. The common manifestations include listeriosis during pregnancy, sepsis and meningitis in newborns, acute febrile gastroenteritis, invasive disease in food-borne outbreaks, and fatal disseminated infection in immunocompromised hosts [1,2]. Unusual localized infections such as pneumonia, hepatitis, arthritis and endophthalmitis have also been reported [2]. The clinical presentation of non-perinatal listeriosis depends on predisposing factors. Patients with a severe immunocompromised status tend to have bacteremia without a focus, localized infection, and central nervous system (CNS) infection with coma or encephalitis [1].

*L. monocytogenes* is a common colonizer of the human gastrointestinal tract with the asymptomatic carrier rate in the general population reported to be 1–10% [3]. The occurrence of invasive listeriosis depends on the integrity of the immune system of the host, and the number of bacteria delivered to the intestinal tract [4,5]. After entering a susceptible host, *L. monocytogenes* adheres to the host receptor E-cadherin [6], is transcytosed across the intestinal epithelium barrier, and then released into the lamina propria by exocytosis [7]. Most of the bacteria become trapped in the liver and spleen by macrophages. In innate immunity, macrophages play an important role in initial control of infection because replication of *L. monocytogenes* occurs primarily within the macrophages that mediate clearance of bacteria. After specific recognition of pathogen-derived products by Toll-like receptors on macrophages, dendritic cells are activated and initiate CD4 and CD8 T cell responses that result in a stable population of *L. monocytogenes*-specific memory T cells [8]. The microorganism can escape immune clearance and re-enter the bloodstream to cause potentially fatal disease unless their replication is controlled by a functional host immune response. Precipitating immunosuppressive agents may lead to the development of invasive disease from latent or subclinical infection.

Listeriosis is mainly transmitted through consumption of contaminated food in the community [9], and vertical transmission from mother to child [10]. Hospital-associated infections usually occur as clustered outbreaks from environmental contact [11-17] or contaminated food [18-25]. Sporadic non-clustered hospital-associated listeriosis is an emerging infectious disease. It has been reported in many countries including the United States [26,27], Europe [1,28,29], Australia [30], Israel [2] and more recently China [31]. It may be triggered from latent or sub-clinical infection by immunosuppressive agents, but the exact pathogenesis and source still needs to be identified. In view of the relatively high rate of asymptomatic carriers of *L. monocytogenes* in the general population [3] and the increasing numbers of patients receiving potent immunosuppressive agents, listeriosis needs to be considered in the differential diagnosis of sepsis in immunocompromised patients.

The current retrospective study of cases at a large teaching medical center in southern Taiwan during a 21-year period was designed to better understand the impact of immunosuppressive agents and underlying diseases in triggering listeriosis, and to compare the clinical features and outcomes of hospital-associated and community-associated listeriosis.

## Methods

### Study design

This retrospective study examined the medical records of patients with listeriosis over a 21-year period from January 1992 to February 2013 at Kaohsiung Veterans General Hospital, a 1200-bed general and tertiary care hospital located in southern Taiwan. We reviewed the data of all patients from whom *L. monocytogenes* was isolated from blood and other sterile sites, including CSF, ascites, pleural effusion and joint fluid. The identified cases were divided into community-associated or hospital-associated infections according to the definitions detailed below. The two groups were then compared according to demographic characteristics, underlying diseases, clinical manifestations, laboratory findings, use of immunosuppressive agents, and outcomes. The clinical manifestations and laboratory findings, including APACHE II score, used in the analysis were recorded at the onset of symptoms that were compatible with listeriosis. The study was approved by the ethics committees in Kaohsiung Veterans General Hospital (VGHKS13-CT11-06).

### Definitions

Listeriosis was indicated when *L. monocytogenes* was isolated from sterile body sites including blood, cerebrospinal or joint fluid of patients with a compatible illness. Hospital-associated cases were patients whose symptoms developed ≥48 hours after admission or within 14 days after discharge for another medical condition. Community-associated cases were patients who presented with signs or symptoms compatible with listeriosis <48 hours after admission. Non-clustered cases were patients unrelated to any other in time or place. Long-term immunosuppressive therapy was receipt of the same immunosuppressive agent for management of an underlying disease for >4 weeks before the development of symptoms related to listeriosis. Precipitating immunosuppressive therapy was receipt of a new immunosuppressive agent or intensified immunosuppressive treatment for the management of disease flare-ups within 4 weeks prior to development of symptoms compatible with listeriosis. CNS infections were meningitis or brain abscesses with abnormal findings on lumbar punctures, but not necessarily with a positive cerebrospinal fluid (CSF) culture. For cases without a positive CSF culture, the diagnosis of CNS infection was based on a positive blood culture with compatible CSF or imaging findings. Listeriosis-related symptoms included fever, reduced consciousness, headache, seizure or nausea, which had no alternative cause. Adequate antibiotic therapy was treatment with penicillin, ampicillin or vancomycin within 24 hours after onset of symptoms related to listeriosis. Mortality was all-cause death within 60 days after the onset of symptoms compatible with listeriosis.

### Statistical analysis

The statistical analyses were performed using SPSS version 12.0 (SPSS Inc., Chicago, IL, USA). Categorical variables were compared using the χ^2^ or Fisher’s exact tests, and non-categorical variables were compared using the Mann–Whitney *U* test. All tests were two-tailed, and *p* < 0.05 was considered significant. Univariable and multivariable analyses of prognostic factors were performed using the LOGISTIC procedure. For the multivariable analysis of mortality, candidate variables were identified as those having a univariate significance of *p* ≤ 0.10, those identified in a previous study, or those believed to be clinically meaningful.

## Results

### Demographic characteristics and underlying diseases of listeriosis patients

**Figure 1:**
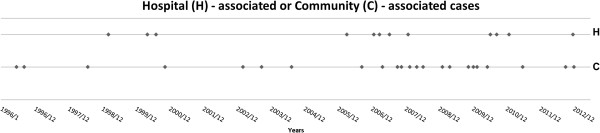
Distribution of 35 cases of listeriosis by calendar year over a period of 21 years.

Thirty-five cases were identified during the 21-year study period (Table 1). All were adults and non-clustered cases (Figure 1). The median age was 61 years (range 20–87 years) and 18 (51.4%) were male. Twelve cases (34.2%) had hospital-associated and 23 (65.7%) had community-associated listeriosis. There were no significant differences in age, sex, or underlying disease between community-associated and hospital-associated cases.

**Table 1:** **Demographic characteristics and underlying diseases among 35 cases of listeriosis**[1]

	**All (*n*** **=** **35)**	**Community-associated (*n*** **=** **23)**	**Hospital-associated (*n*** **=** **12)**	***p*-value**
Age, median years (range)	61 (20–87)	64 (29–87)	68 (21–81)	0.29
Male sex (%)	18 (51.4)	11 (47.8)	7 (58.3)	0.56
Diabetes mellitus (%)	10 (28.6)	8 (34.8)	2 (16.7)	0.43
Chronic kidney disease (%)	12 (34.3)	10 (43.4)	2 (16.7)	0.15
Hematologic malignancy (%)	5 (14.3)	2 (8.6)	3 (25.0)	0.31
Solid tumor (%)	12 (28.6)	4 (17.4)	6 (50)	0.06
Autoimmune disease (%)	8 (22.9)	6 (26.1)	2 (16.7)	0.69
Immunosuppressive agents (%)	18 (51.4)	7 (30.4)	11 (91.7)	< 0.01
Long-term immunosuppressive agents^a^ (%)	8 (22.9)	7 (30.4)	1 (8.3)	0.22
Precipitating immunosuppressive agents^b^ (%)	12 (34.3)	1 (4.3)	11 (91.7)	< 0.01
Chronic obstructive pulmonary disease (%)	3 (8.6)	1 (4.3)	2 (16.7)	0.27
Liver cirrhosis (%)	6 (17.1)	5 (21.7)	1 (8.3)	0.64
Peptic ulcer disease (%) [32]	7 (20)	7 (30.4)	0	0.07

Data are no. (%) of patients, unless otherwise indicated.

^a^These included corticosteroids alone in 5; corticosteroids plus a biological agent in 1; and corticosteroids plus azathioprine in 2.

^b^These included high doses of corticosteroids and chemotherapy.

### Immunocompromised status of listeriosis patients

Thirty-two of the 35 (91.4%) patients were immunocompromised. Many of these patients had several diseases associated with immune deficiency (Table 1). These included neoplastic, autoimmune and steroid-dependent obstructive pulmonary diseases, alone or in combination with other diseases such as diabetes, chronic renal failure, and hepatic cirrhosis. No patients had HIV infection or organ transplantation. Eighteen patients (51.4%) received one or more immunosuppressive agents (Table 1). Eight (44.4%) of these 18 patients received long-term immunosuppressive agents, 12 (66.7%) received a precipitating immunosuppressive agent, and two (11.1%) received both long-term and precipitating immunosuppressive agents. Of those receiving a precipitating immunosuppressive agent, six (50%) patients received a corticosteroid and six (50%) received both a corticosteroid and chemotherapy. Hospital-associated cases were significantly more likely than community-associated cases to have received a precipitating immunosuppressive agent (n = 11, 91.7% vs. n = 1, 4.3%, *p* < 0.01).

### Clinical and laboratory manifestations of listeriosis

**Figure 2:**
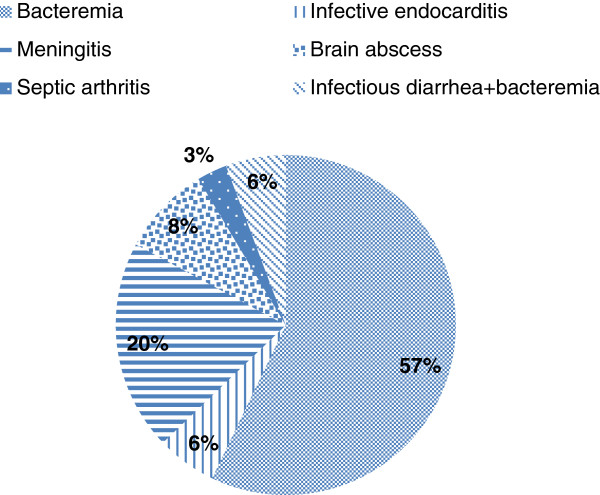
**Clinical syndromes of the 35 cases of listeriosis.** The majority (n = 20, 57%) presented as primary bacteremia without obvious site of involvement. Central nervous system involvement (n = 10, 28.6%) was the next most common. Other clinical syndromes included infective endocarditis (n = 2, 6%), infectious diarrhea (n = 2, 6%), and septic arthritis (n = 1, 3%).

The clinical and laboratory findings of the 35 cases of listeriosis are summarized in Table 2 and Figure 2. *L. monocytogenes* was isolated from blood cultures in 33 of the 35 (94.2%) patients. The other two cases with negative blood cultures had *L. monocytogenes* isolated from joint fluid or from CSF. Among the 10 patients with CNS involvement, the organism was isolated from the initial CSF in six (60%) and from blood in nine (90%). Most of the 35 patients were febrile (88.6%) and 37.1% had symptoms of CNS involvement. These included altered consciousness, seizure and focal neurological findings. There were no other significant differences between the groups in clinical or laboratory findings.

**Table 2:** **Clinical manifestations**, **laboratory findings and outcomes among 35 cases of listeriosis**

	**All ( *n * = 35)**	**Community-associated (*n * = 23)**	**Hospital-associated (*n * = 12)**	***p*-value**
Clinical findings				
APACHE II score, median (range)	15 (4–46)	12 (4–46)	19 (6–30)	0.55
Headache (%)	4 (11.4)	3 (13)	1 (8.3)	1.0
Nausea (%)	3 (8.6)	1 (4.3)	2 (16.7)	0.27
Reduced consciousness (%)	11 (31.4)	6 (26.1)	5 (41.7)	0.45
Seizure (%)	6 (17.1)	3 (13)	3 (25)	0.39
Focal neurologic (%)	3 (8.6)	2 (8.7)	1 (8.3)	1.0
Diarrhea (%)	3 (8.6)	2 (8.7)	1 (8.3)	1.0
Fever (%)	31 (88.6)	20 (87)	11 (91.7)	1.0
Adequate antibiotic therapy within 24 hours (%)	4 (11.4)	1 (4.3)	3 (25)	0.16
CNS infection (%)	10 (28.6)	8 (34.8)	2 (16.7)	0.43
60-day mortality (%)	12 (34.3)	4 (17.4)	8 (66.7)	0.01
Hospital duration, median day (range)	28 (1–126)	20 (1–124)	53 (15–126)	0.01
Laboratory findings				
ALT, median IU/L (range)	41 (6–248)	47.5 (6–248)	39 (15–177)	0.88
Total bilirubin, median mg/dL (range)	0.9 (0.2–7.9)	0.8 (0.2–4)	0.5 (0.2–7.9)	0.24
Albumin, median g/dL (range)	2.7 (1.4–3.9)	2.6 (1.7–3.8)	2.4 (1.4–3.9)	0.93
White blood cells, median 10^9^/L (range)	8.38 (0.19–28.16)	13.39 (3.36–27.47)	8.38 (0.19–28.16)	0.06
Platelets, median /mm^3^ (range)	117 (4–350)	158.72 (27.7–350)	101.91 (4–275)	0.18
Serum creatinine, median mg/dL (range)	1.1 (0.4–17.2)	1.10 (0.58–17.2)	1.10 (0.4–8.84)	0.07
Site of *L. monocytogenes* isolates				
Blood (%)	33 (94.3)	21 (91.3)	12 (100)	0.54
CSF (%)	6 (17.1)	5 (21.7)	1 (8.3)	0.64
Joint fluid (%)	1 (2.8)	1 (4.3)	0 (0)	1.0

Data are no. (%) of patients, unless otherwise indicated. APACHE II, Acute Physiology And Chronic Health Evaluation II; ALT, alanine aminotransferase.

The clinical presentation of the 35 cases of listeriosis is shown in Figure 2. The most common clinical syndrome was primary bacteremia in 20 (57%) patients. Ten (28%) patients had CNS involvement, seven with meningitis and three with a brain abscess. Localized infection presenting as septic arthritis of the knee was noted in one case (2.9%).

### Outcomes

Twelve of the 35 patients (34.2%) died, all as a direct result of listeriosis. Hospital-associated cases had a longer hospital stay (53 vs. 20 days, *p* = 0.01) and a greater 60-day mortality rate (66.7 vs. 17.4%, *p* = 0.01) than community-associated cases (Table 2). In multivariable analysis of 60-day mortality, an APACHE II score > 21 [odds ratio (OR), 40.43; 95% confidence interval (CI), 1.07-1515.00; *p* = 0.04] and receipt of a precipitating immunosuppressive agent within 4 weeks (OR, 285.81; 95% CI, 2.34–34859.49; *p* = 0.02) were prognostic indicators of poor survival (Table 3). Four of the 35 (11.4%) cases were treated with an appropriate antibiotic within 24 hours following the onset of the listeriosis-related symptoms. Two were treated with penicillin for suspected pneumonia; one with ampicillin and gentamicin for lymphocytic meningitis; and one patient received vancomycin for suspected recurrence of methicillin-resistant *Staphylococcus aureus* bacteremia.

**Table 3:** **Multivariable analysis of 60**-**day mortality in the 35 adult patients with listeriosis**

**Variables**	**Odds ratio**	**95% confidence interval**	***p*-value**
Age	0.94	0.847–1.049	0.28
Sex (male)	7.26	0.118–446.821	0.35
Chronic kidney disease	1.79	0.068–46.805	0.73
Autoimmune disease	0.18	0.001–21.830	0.48
Precipitating immunosuppressive agents^a^	285.82	2.343–34 859.494	0.02
CNS involvement	15.01	0.166–1362.565	0.24
APACHE II >21	40.44	1.079–1515.001	0.04
ALT	1.03	0.998–1.059	0.06
Adequate antibiotic agents	389.72	0.241–631 352.978	0.11

^a^Defined in methods.

### Clinical characteristics and precipitating immunosuppressive agents in 12 sporadic non-clustered cases of hospital-associated listeriosis

Detailed information about the underlying diseases, cause of admission, clinical syndromes of listeriosis, and reason for precipitating immunosuppressive therapy for the 12 hospital-associated cases of listeriosis are shown in Table 4. None of these cases showed clustering according to time or place (Figure 1). Listeriosis-related symptoms appeared at a median of 24 days (range, 10–59 days) following admission. Ten (83.3%) of the patients had primary bacteremia and two (16.7%) had meningitis with bacteremia. Eleven of the 12 (91.7%) patients had received a precipitating immunosuppressive agent within 4 weeks prior to the onset of listeriosis. Five of the 11 (45.4%) patients received corticosteroids at an equivalent dose of prednisolone ≥1 mg/kg, and six (54.5%) patients received both corticosteroids and chemotherapy. The median time from the physician’s order for a precipitating immunosuppressive agent to the onset of listeriosis-related symptoms was 12 days (range, 4–27 days). One of the patients who did not receive a precipitating immunosuppressive agent had a severe underlying disease. This was a left adrenal gland tumor with invasion of the tail of the pancreas and multiple bone metastasis. It is possible that the tumor may have damaged the integrity of his gastrointestinal tract, leading to invasion with *L. monocytogenes*.

**Table 4:** **Clinical characteristics and detailed history of precipitating immunosuppressive agents in 12 sporadic non-clustered cases of hospital**-**associated listeriosis**

**Case**	**Underlying disease**	**Cause of admission**	**D 1**^**a**^	**Clinical syndrome of listeriosis**	**Type of precipitating immunosuppressive agent**	**D 2**^**b**^	**Cause of precipitating immunosuppressive agent**
1	Breast cancer with liver and lung metastasis	Scheduled chemotherapy	10	Primary bacteremia	Corticosteroid and chemotherapy with epirubicin and paclitaxel	9	Scheduled chemotherapy
2	Hepatic cell carcinoma with multiple bone metastasis	Management of low back pain	14	Primary bacteremia	Corticosteroid	7	Management of paraplegia caused by hepatic cell carcinoma with spine metastasis
3	Systemic lupus erythematosus	Management of pregnancy complicated with pre-eclampsia	59	Meningitis and bacteremia	Corticosteroid and chemotherapy with cyclophosphamide	17	Pulse therapy for lupus nephritis with flare-up
4	Lymphocytic myocarditis complicated with congestive heart failure	Management of acute pulmonary edema	59	Meningitis and bacteremia	Corticosteroid and chemotherapy with cyclophosphamide	23	Pulse therapy for lymphocytic myocarditis
5	New diagnosis of pancreatic cancer with lung metastasis	Evaluation of lung mass	30	Primary bacteremia	Corticosteroid and chemotherapy with cyclophosphamide	22	For suspected Wegner’s granuloma
6	New diagnosis of left adrenal gland with pancreas tail invasion and multiple bone metastasis	Evaluation of abdominal pain	42	Primary bacteremia	Nil	Nil	
7	Lung cancer	Management of obstructive pneumonia	18	Primary bacteremia	Corticosteroid	4	Management of paraplegia caused by lung cancer with spine metastasis
8	New diagnosis of acute lymphoblastic leukemia	Evaluation of petechiae and bruising over extremity	10	Primary bacteremia	Corticosteroid and chemotherapy with cystarabine, methotrexate, vincristine and asparaginase	11	Induction chemotherapy for acute lymphoblastic leukemia
9	Ischemic heart disease complicated with congestive heart failure	Management of acute pulmonary edema caused by fluid overload	13	Primary bacteremia	Corticosteroid	12	For suspected superimposed chronic obstructive pulmonary disease with flare-up
10	New diagnosis of multiple myeloma	Evalaution of oliguria	51	Primary bacteremia	Corticosteroid	27	Management of paraplegia caused by multiple myeloma with spinal compression
11	Diffuse large B cell lymphoma	Scheduled chemotherapy	11	Primary bacteremia	Corticosteroid and chemotherapy with methotrexate, rituximab and gemcitabine	10	Scheduled chemotherapy
12	Nasopharyngeal carcinoma with skull bone metastasis	Management of hypercalcemia caused by malignancy	52	Primary bacteremia	Corticosteroid	14	Management of drug allergy caused by nonsteroidal anti-inflammatory drug

^a^Duration 1: from admission to onset of symptoms related to listeriosis (days).

^b^Duration 2: from prescription of precipitating immunosuppressive agent to onset of symptoms related to listeriosis (days).

## Discussion

Sporadic, non-clustered, hospital-associated listeriosis is a relatively rare, but life-threatening disease. We identified 12 cases in adults at a large medical center in southern Taiwan over a period of 21 years, or 0.34 cases per year. In a similar study conducted at a large tertiary care hospital in China, Yang et al. found nine non-clustered hospital-associated cases over a period of 12 years, or 0.75 cases per year [31]. We also identified 23 community-associated cases. In the present study, 32 of the 35 cases (91.4%) were immunocompromised. This is similar to previous reports [1,28,30,33]. The listeriosis-related mortality rate of 34.3% observed in the current study is similar to other reports (24–52%) [2]. The 60-day mortality rate was significantly higher in patients with hospital-associated than community-associated listeriosis (66.7% vs. 17.4%, *p* = 0.007). Other reports showed a mortality rate of hospital-associated listeriosis of 27.2–53% [30,31].

Although it may be argued that a hospital-associated case defined by symptoms developing ≥48 hours after admission could be a case of community-associated listeriosis with a protracted course and delayed diagnosis, detailed analysis of the cause of admission and initiation of a precipitating immunosuppressive agent in these 12 hospital-associated cases suggests these were truly hospital-associated cases rather than community-associated with a subacute course.

Reactivation of latent or subclinical infection by immunosuppressive agents provides an attractive explanation for the mechanism of sporadic non-clustered hospital-associated listeriosis in immunocompromised hosts. In the present study, almost all of the non-clustered hospital-associated cases (n = 11, 91.7%) received a new or more intensive precipitating immunosuppressive agent within 4 weeks prior to the onset of listeriosis compared with only 4.3% (n = 1) of the community-associated cases (*p* < 0.001). Other investigators have reported the occurrence of listeriosis within 4 weeks after receiving chemotherapy or corticosteroids in patients with underlying malignancies [2,33]. Listeriosis has also been reported among patients undergoing therapy with biological agents such as infliximab [26,27,29] and etanercept [34]. This concept that immunosuppressive agents trigger latent listeriosis is further supported by the relatively short time interval (median 12 days; range, 4–27 days) from the order for a precipitating immunosuppressive agent to onset of severe, life-threatening hospital-associated listeriosis.

In the current study, only four of the 35 (11.4%) cases were treated with an appropriate antibiotic within 24 hours after the onset of listeriosis. The rate was low for both community-associated and hospital-associated cases (4.3% and 25%, *p* = 0.16). This may arise because of the non-specific manifestations and rare occurrence of listeriosis, combined with the primary physician being unfamiliar with the disease. Education of physicians about the presentation of listeriosis, especially sepsis and meningitis in the elderly and immunocompromised patients, and in the setting of new or increased treatment with immunosuppressive agents in hospital may improve the rate of appropriate prescribing of antimicrobial agents.

Mortality rates from listeriosis tend to be higher in severely immunocompromised patients, such as those with AIDS, or who receive immunosuppressive therapy for organ transplantation, than in less immunocompromised patients or apparently healthy hosts [1]. In the multivariable analysis of mortality we found that an APACHE II score > 21 and receipt of precipitating immunosuppressive agents within 4 weeks prior to the onset of listeriosis were indicators of poor prognosis. Other factors reported in the literature for poor prognosis include hepatic decompensation [33], renal failure, and age >65 years [35].

Prophylaxis with trimethoprim-sulfamethoxazole (TMP-SMZ) for *Pneumocystis jiroveci* and *Toxoplasma gondii* is given in patients with advanced human immunodeficiency virus (HIV) infection and organ transplant recipients [36-38]. In the current study, no patients had HIV infection or organ transplantation and they didn’t receive TMP-SMZ prophylaxis at the onset of listeriosis. Mark et al. revealed that prescription of TMP-SMZ for *Pneumocystis jiroveci* prophylaxis in persons with HIV infection also significantly decrease risk for other infections, including *Haemophilus*, *Salmonella*, and *S. aureus* disease, but not listeriosis [39]. However, estimation of TMP-SMZ prophylaxis for listeriosis in HIV-infected patient and other hosts with a severe immunocompromised status needs further studies.

The strengths of the current study are the relatively large number of cases of a rare disease and our ability to determine the key role of precipitating immunosuppressive agents as the major risk factor for life-threatening sporadic non-clustered hospital-associated listeriosis. The current study was conducted in a single institution in Taiwan, but the findings are consistent with reports from many other countries. The weakness is the retrospective nature of this long-term study. However, this approach was the only way we could collect a sufficient number of cases for detailed analysis. It prevented us from being able to identify the potential source(s) of infection, detect carriers, preserve the bacterial isolates for molecular epidemiological studies and to evaluate the efficacy of antimicrobial therapy. Listeriosis is not a notifiable disease in Taiwan. Therefore relevant data about the epidemiology and clinical manifestations are limited [33,40-43].

## Conclusions

Sporadic non-clustered hospital-associated listeriosis is an emerging infectious disease in immunocompromised hosts. It should be included in the differential diagnosis of sepsis, particularly in those receiving new or increased doses of immunosuppressive agents, such as corticosteroids or chemotherapy within the previous 4 weeks. Whether the incidence of this disease will increase in association with the development and widespread use of new and more focused immunosuppressive agents needs further study.

## Abbreviations

APACHE II: Acute Physiology and Chronic Health Evaluation II; ALT: alanine aminotransferase; CI: Confidence interval; CNS: Central nervous system; CSF: Cerebrospinal fluid; HIV: Human immunodeficiency virus; OR: Odds ratio; TMP-SMZ: Trimethoprim-sulfamethoxazole.

## Competing interests

The authors declare that they have no competing interests.

## Authors’ contributions

All authors were responsible for the conception and design of the study, analysis and interpretation of the data, drafting and critical revision of the manuscript and final approval of the manuscript.

## Pre-publication history

The pre-publication history for this paper can be accessed here:

http://www.biomedcentral.com/1471-2334/14/145/prepub
